# Virus and host-specific differences in oral human herpesvirus shedding kinetics among Ugandan women and children

**DOI:** 10.1038/s41598-017-12994-0

**Published:** 2017-10-12

**Authors:** Laura Matrajt, Soren Gantt, Bryan T. Mayer, Elizabeth M. Krantz, Jackson Orem, Anna Wald, Lawrence Corey, Joshua T. Schiffer, Corey Casper

**Affiliations:** 1 Fred Hutchinson Cancer Research Center, Seattle, WA USA; 2 Department of Pediatrics, Division of Infectious Diseases, University of British Columbia, Vancouver, BC Canada; 3 Uganda Cancer Institute, Makerere University, Kampala, Uganda; 4 College of Health Sciences, Makerere University, Kampala, Uganda; 5 Department of Medicine, University of Washington, 98195 Seattle, WA USA; 6 Department of Epidemiology, University of Washington, Seattle, WA USA; 7 Department of Laboratory Medicine, University of Washington, Seattle, WA USA; 8 Clinical Research Division, Fred Hutchinson Cancer Research Center, Seattle, WA USA; 9 Infectious Disease Research Institute, Seattle, WA USA

## Abstract

Human herpesviruses (HHV) establish lifelong latent infection and are transmitted primarily via shedding at mucosal surfaces. Each HHV causes a unique spectrum of disease depending on the infected individual’s age and immunity. We collected weekly oral swabs from young children and mothers in 32 Ugandan households for a median of one year. We characterized kinetics of oral shedding during primary and chronic infection for each virus. Cytomegalovirus (CMV), Epstein-Barr virus (EBV), and HHV-6 were shed at high rates following primary infection. The rate of oral herpes simplex virus (HSV) shedding was lower overall, and children and mothers with chronic HSV infection had lower shedding rates than children with primary infection. CMV shedding rate and viral load were higher in children with primary infection compared to children with chronic infection, and even lower in mothers with chronic infection. HHV-6 shedding rate and viral load were similar between children with primary or chronic infection, but lower in mothers. EBV shedding rate and quantity decreased less dramatically in mothers versus children, with HIV-positive mothers shedding at a higher rate than HIV-negative mothers. Each HHV has a distinct pattern of oral shedding which depends partially on the age and immune status of the host.

## Introduction

The human herpesviruses (HHVs) are DNA viruses that establish latency, allowing lifelong infection. Periodic viral reactivation results in mucosal shedding, by which transmission to new hosts can occur. Each HHV causes unique manifestations, which reflect virus-specific differences in cell tropism and patterns of replication during primary infection and reactivation, as well as the immune status of the host. Primary HHV infections frequently occur during early childhood, particularly in the developing world^1–4^. While levels of HHV replication and mucosal shedding are generally greater in young children and during primary infection^4–8^, there are limited data that directly contrast shedding patterns in children versus adults. In this study, we utilized a detailed longitudinal cohort to compare the oral shedding patterns of multiple HHVs – herpes simplex virus (HSV), Epstein-Barr virus (EBV), cytomegalovirus (CMV), and HHV-6 – among young children with primary infection, older children with chronic infection, and their mothers to investigate the natural history of mucosal replication by different HHVs. These results illustrate that the pattern of oral shedding is unique for each HHV and depends on the timing of infection and immune status of the infected individual.

## Results

### Study participants and infection status

Thirty-two mothers, 32 newborns and 49 secondary children were followed for a median duration of 57 weeks. Two secondary children were excluded because they had fewer than 2 swabs collected. The median age for the remaining 47 secondary children was 3.5 years (range 0.3–6.3 years). Seventeen mothers, four secondary children and no infants were HIV-infected at the time of enrollment; seven secondary children had unknown HIV status.

Postnatal EBV, CMV and HHV-6 acquisition occurred in a majority of infants, whereas HSV acquisition was less common (Table 1). Congenital CMV infections occurred in two infants, who were excluded from the analysis of CMV shedding. Combining serology and shedding data, we identified three primary EBV infections among secondary children (at ages 1.7, 1.8, and 2.9 years). We also identified one secondary child with primary HSV-1 infection at 4.4 years of age, based on seroconversion. No other HHV infection in secondary children was deemed to be primary by our criteria which are described in the Methods. Two secondary children were uninfected with CMV, 13 uninfected with HSV, and 8 uninfected with HHV-6. These children were excluded from shedding analyses for those viruses. All mothers had serologic evidence of past CMV infection. All but two mothers had serological evidence of past HSV-1 infection; one mother was HSV seronegative and excluded from HSV analyses and one mother had insufficient quantity of sample for serologic testing but did have at least two HSV-1 qPCR positive oral swabs and so was retained in HSV analyses. We originally tested oral samples for HHV-8 but observed minimal shedding in infants or secondary children so excluded this virus from the analysis.

**Table 1:** Study participants and samples.

	HSV	EBV	CMV^a^	HHV-6
**Primary infections, n** (**%**)	10 (31%)^b^	22 (69%)^c^	20 (67%)	24 (75%)
Age in months, median (range)	0.03 (0.03–47)	0.1 (0–25)	0.1 (0–0.3)	0.07 (0–0.3)
Boys, %	50	50	50	46
Percentage of positive swabs^d^	39	95	98	87
**Chronic infections in children, n** (**%**)^e^	33 (67%)	44 (90%)	42 (95%)	39
Age in years, median (range)	3.5 (1.3–6.3)	3.6 (0.3–6.3)	3.5 (0.3–6.3)	3.5 (0.3–6.3)
Boys (%)	48	50	48	46
Percentage of positive swabs	17	79	75	94
**Mothers** (**n**)^**f**^	31	32	30	32
HIV infection (%)	55	53	50	53
Percentage of positive swabs	7	73	24	51

^a^Two infants had congenital CMV infection. These households were excluded from analyses of CMV.

^b^Nine primary HSV infections were type 1 (8 from infants and 1 from a secondary child) and one was type 2 (infant).

^c^Three primary EBV infections were identified among secondary children.

^d^Percentage of positive swabs was computed after infection for primary infected children and over the duration of the study for chronically infected children and mothers.

^e^Chronic infection in secondary children was assumed for all children not meeting criteria for primary infection and not classified as uninfected. Two secondary children were uninfected with CMV, 13 uninfected with HSV, and 8 uninfected with HHV-6; these children were excluded from analyses of these viruses and do not appear in Table 1. Full classification procedure is given in Supp. Figure 1.

^f^All mothers had serologically-confirmed chronic infection with CMV. All mothers shown in Table 1 had either serologically-confirmed chronic infection with HSV-1 (n = 30) or at least two HSV-1 PCR positive swabs in the absence of serology data (n = 1). Chronic infection with EBV and HHV-6 was assumed for all mothers.

All primary infections were asymptomatic. We did not assess chronically infected children or mothers for symptoms.

### General shedding patterns

**Figure 1:**
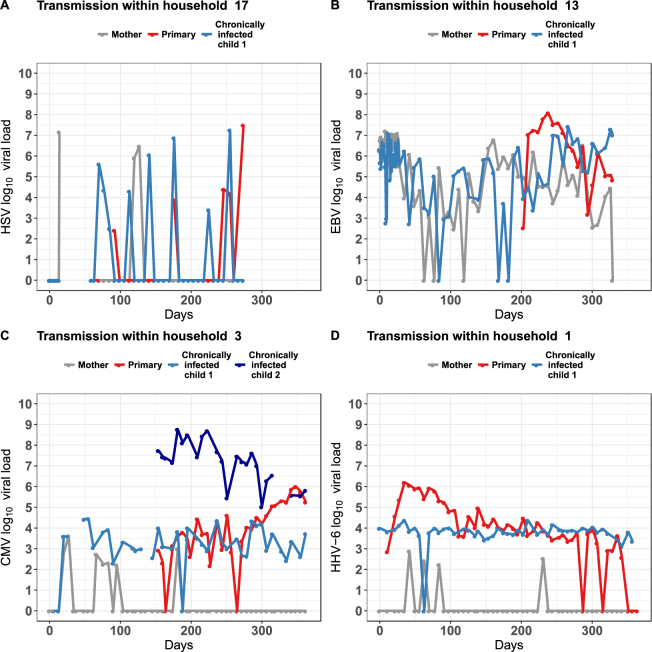
Representative oral human herpesvirus shedding patterns within families. Each panel depicts the oral shedding of one virus in a single household. The weekly viral load of (**A**) HSV, (**B**) EBV, (**C**) CMV, and (**D**) HHV-6 detected in oral swabs by qPCR is shown for each family member (mother, gray; children with primary infection, red; children with chronic infection, blue). Missing data points are not included as points on the graphs. Complete sets of plots for all the families participating in the study can be found in the Supplementary Figures S1–S4.

Primary infection in children had a notable pattern of persistent shedding over time and in higher quantities when compared to the shedding of the same virus by mothers or children with chronic infection in the same household. The nature of HSV shedding was episodic in infants, children with chronic infecion and mothers, with intermitent negative samples frequently noted, most commonly after only 1–2 consecutive positive samples (Figs 1 and S1). EBV displayed a mixed pattern in all three age groups with instances of episodic shedding lasting only a single week and more continuous shedding persisting for many months; in general, viral loads were only somewhat stable during sustained EBV shedding and often were similar across age cohorts (Figs 1 and S2). As previously described, CMV shedding in infants consisted of occasional brief episodic shedding followed by sustained shedding at high viral loads with notable expansion, stabilization and contraction phases of shedding^9,10^; instances of prolonged, continuous shedding, as well as brief, episodic CMV shedding were noted in chronically infected children and mothers; within each family, viral loads in chronically infected children and mothers were consistently lower than those observed during primary infection (Figs 1 and S3). HHV-6 shedding was continuous in both infants and chronically infected children, but usually episodic and sometimes at lower viral loads in mothers (Figs 1 and S4).

**Figure 2:**
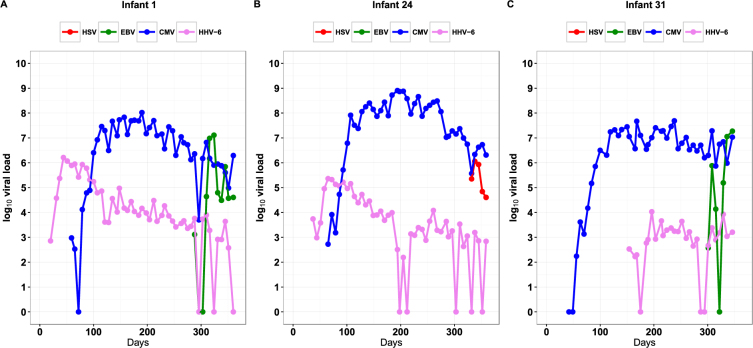
Representative examples of shedding patterns of children with primary infections with multiple human herpesviruses. Beginning at the time of primary infection, shown is the quantity of viral DNA detected in oral swabs collected weekly from the time of birth (Day 0). Each panel shows data from one representative infant. Missing data points are not included as points on the graphs. Complete sets of primary infections can be found in the Supplementary Figure S5.

Due to the high incidence and prolonged shedding of primary HHV infections, many infants demonstrated concurrent shedding of multiple viruses (Figs 2 and S5). During the first 365 days of life at any point in time, one infant shed a single virus, four infants shed two viruses, seventeen infants shed three viruses, and ten infants shed four viruses. All infants who shed 3 or 4 HHVs at any point during the first year of life, also shed these viruses concurrently at contemporaneous timepoints.

We describe unique shedding patterns for each virus below and make comparisons across age cohorts within virus, and between viruses within each age cohort. All shedding rate comparisons are summarized in Supplementary Table 1 while all viral load comparisons are summarized in Supplementary Table 2.

### Unique age dependent HSV shedding rates and viral loads

**Figure 3:**
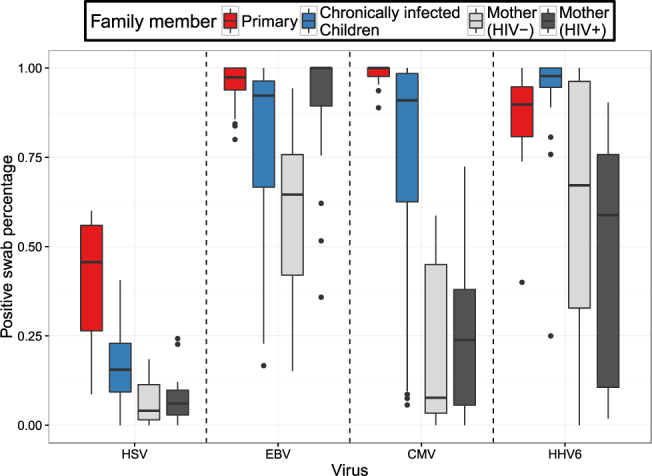
Proportions of positive swabs according to age group for each human herpes virus. Boxplots represent distributions of individual values. The boxes represent the interquartile range. The stems in the plots represent up to 1.5 times the value of this range.

There was a higher HSV shedding rate in primary infected children relative to children with chronic infection (Figs 3 and S6A). HIV-negative and positive mothers in turn shed HSV less frequently than secondary children (Figs 3 and S6A).

The median HSV shedding rate during primary HSV was comparatively low relative to other primary HHVs, though HSV was still detected in nearly 50% of samples during primary infection; a similar trend was noted for HSV shedding relative to other HHVs among the secondary children (Figs 3 and S6B),. HIV-negative and positive mothers also shed HSV at lower rates than EBV and HHV-6 (Figs 3 and S6B).

**Figure 4:**
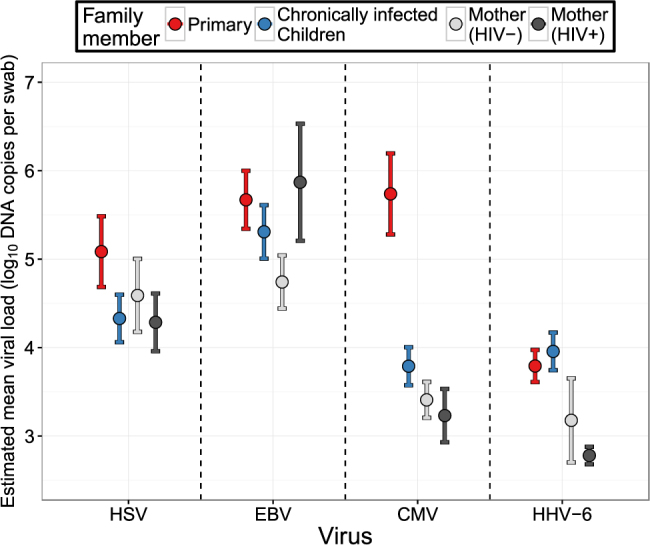
Estimated mean viral load for positgive samples according to age group for each human herpesvirus. The bars represent 95% confidence intervals.

HSV viral loads were higher in children with primary infection relative to chronically infected children and HIV negative and positive mothers with chronic infection who had equivalent mean viral loads; however, these differences did not reach statistical significance (Figs 4 and S7A).

### Unique age dependent EBV shedding rates and viral loads

EBV shedding rates were extremely high in all age cohorts. Children with chronic EBV infection had statistically significantly lower shedding rates than children with primary EBV infections who shed nearly constantly (Figs 3 and S6A). HIV-negative mothers shed EBV at very high rates (median 65%) that were not statistically lower than rates in secondary children. There was considerable variability in the rate of EBV shedding among children with chronic infection and among mothers relative to primary infections (Fig. 3).

Similar trends were noted for EBV viral loads which were highest during primary infection with lower mean viral load in secondary children and lowest mean viral load in HIV-negative mothers; the only statistically significant difference in mean EBV viral load was between primary infected children and HIV negative mothers (Figs 4 and S7A).

EBV viral loads were notably high in secondary children relative to all other HHVs in this age cohort, and in HIV negative mothers relative to CMV and HHV6 (Figs 4 and S7B).

### Unique age dependent CMV shedding rates and viral loads

Children with primary CMV infection shed at extremely high rates (median 100%) and had statistically significantly higher shedding rates than secondary children (Figs 3 and S6A). However, the drop off in shedding rates between secondary children and HIV-negative and positive mothers was more profound for CMV than for other viruses (Figs 3 and S6A). There was also greater variability in the rate of CMV shedding among children with chronic infection and mothers relative to primary infections (Fig. 3). While secondary children shed CMV at similar rates to EBV and HHV-6, maternal CMV shedding rates were statistically lower than maternal EBV and HHV-6 shedding rates (Figs 3 and S6B).

There was a dramatic, strongly significant decrease in viral load (almost 2 log_10_ DNA copies per swab) between children with primary versus chronic CMV infection, with no differences noted between children with chronic infection and mothers (Figs 4 and S7A). HIV negative mothers shed CMV at lower viral loads than HSV and EBV Figs 4 and S7B).

### Unique age dependent HHV-6 shedding rates and viral loads

Unlike, the other HHVs, there was no drop off in shedding rate of HHV-6 between primary infection and secondary children: both groups shed at extremely high rates (Figs 3 and S6A). Maternal HHV-6 shedding rates showed great heterogeneity though median rates were high in both HIV-1 negative and positive cohorts with significantly lower rates in HIV-positive mothers relative to both age cohorts in children (Figs 3 and S6A).

In contrast to the other HHVs, children with primary and chronic infection, and HIV negative mothers, shed HHV-6 at equivalent viral loads (Figs 4 and S7A). HHV-6 viral loads during primary infection were lower than all other HHVs (Figs 4 and S7B).

### Different EBV shedding rates and viral loads among HIV-positive and -negative mothers

There were no statistically significant differences according to shedding rate (Figs 3 and S6A) or viral load (Figs 4 and S6A) between HIV negative and positive mothers for HSV,CMV or HHV-6. Only EBV infection had a statistically significant higher oral shedding rate in HIV-positive versus HIV-negative mothers (Figs 3 and S6A). EBV mean viral loads appeared higher in HIV positive mothers relative to HIV negative mothers though this trend did not reach statistical significance (Figs 4 and S7A). HHV-6 shedding rate and viral load did not statistically differ between HIV-negative and positive mothers, but median shedding rate and mean viral load were lower in the HIV-positive mothers relative to children with primary and chronic infection.

### Summary of differences in shedding by cohort group and virus

**Figure 5:**
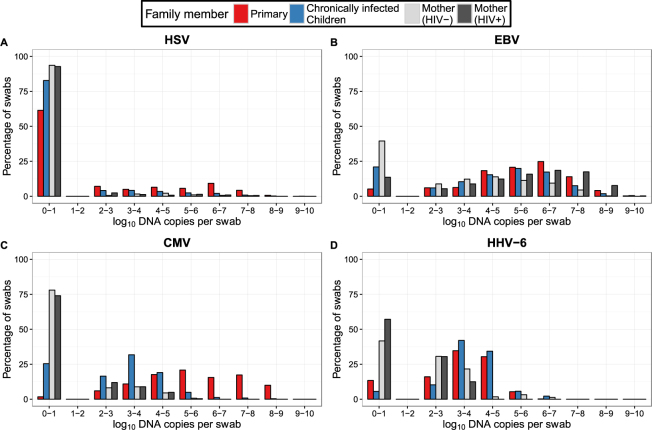
Summary of shedding rate and quantity across viruses and cohort groups. Mothers are classified according to their HIV status. Frequency histograms demonstrate unique shedding patterns for (**A**) HSV, (**B**) EBV, (**C**) CMV and (**D**) HHV-6.

We used frequency histograms to simultaneously display shedding rate and quantity, highlighting general trends of shedding specific for each virus (Fig. 5). In general, HSV was notable for lower shedding rates than the other viruses in children with primary infection with a decrease in shedding rate at all viral loads in children with chronic infection (Fig. 5A). EBV was shed frequently by all cohort groups; changes according to age were subtle though HIV-negative mothers shed less frequently and at lower viral loads than chronically infected children or HIV-positive mothers; HIV-positive mothers shed at the highest viral loads, at rates akin to children with primary infection (Fig. 5B). CMV displayed a highly distinct pattern with a small change in shedding rate between children with primary and chronic infection, but a profound decrease in mean viral load; mothers had a lower shedding rate overall than children with chronic infection (Fig. 5C). HHV-6 shedding rates were generally high but viral loads were lower than all other viruses; HHV-6 was also unique because shedding kinetics were comparable in children with primary or chronic infection, while viral load and shedding rates were lower in mothers (Fig. 5D).

## Discussion

The distinct shedding patterns of different HHVs allow inferences about the balance between viral replication and host immunity as infection progresses from acute to chronic phase. While HHV shedding is generally more common and at higher viral loads following primary infection, a more detailed assessment of individual HHVs has not been performed^4–8^. Here we demonstrate that in a cohort of Ugandan families sampled weekly for a median of over a year, patterns of shedding kinetics were unique for each of the HHVs studied. In particular, shedding rates and viral loads showed distinctive trends as a function of age. HIV infection appeared to only increase oral EBV shedding in mothers. These results imply that each HHV elicits mucosal immune responses that differ dramatically in terms of intensity and timing.

EBV, CMV and HHV-6 were shed nearly constantly during primary infection in infancy and throughout early childhood, while the HSV shedding rate was approximately 50%. Concurrent primary infection was extremely common. This frequent oral replication of multiple HHVs has little overt impact on health or symptoms^4^, underscoring how well each virus has co-adapted with its human host; viral replication and immune control are compartmentalized such that mucosal shedding is frequent but systemic viral spread and end-organ damage is prevented^11–13^. CMV, EBV and HSV viral loads were higher than those of HHV-6 during primary infection. We speculate that differences in viral load among HHVs reflect underlying heterogeneity in rates of viral replication and spread, the effectiveness of the local immune response, and perhaps target cell availability^10,14,15^.

Age-dependent control of viral replication and disease manifestations is well recognized for some HHVs^16–18^. By examining a cohort that ranged from birth to adulthood, we quantified profound differences in the dynamics of oral shedding among these viruses. The HSV shedding rate was markedly lower among children with chronic infection compared with primary infection, though the distribution of viral loads decreased less substantially and shedding was episodic in both groups. The HSV shedding rate and viral load were lower still in mothers versus chronically infected children, suggesting that intensification of immunity continues even after resolution of primary infection.

EBV demonstrated an altogether different shedding structure. Overall, shedding rates were considerably higher than HSV. While the shedding rate decreased in a significant stepwise fashion from infancy to childhood, and viral load continued to decrease into adulthood, marked heterogeneity was seen among children and mothers with shedding rates exceeding 50% in over half of both cohorts. On the other hand, as with HHV-6, age-associated decreases in viral load were less impressive relative to CMV and HSV.

Shedding patterns of CMV contrasted starkly with those of HSV and EBV. The shedding rate of CMV decreased markedly in some children with chronic infection relative to primary infection, but many chronically infected children shed CMV nearly constantly akin to primary infection. Yet, CMV viral loads were dramatically lower in children with chronic versus primary infection. Mothers in our cohort had marginally lower viral loads than children with chronic infection, but had a considerably lower shedding rate and a higher likelihood of episodic shedding. Recent analyses by our group suggest that maternal oral shedding may represent re-infection via the neonate rather than reactivation. Overall, these results demonstrate ongoing fortification of the anti-CMV immune response from childhood into adulthood, perhaps by a different mechanism than occurs with transition from infancy to childhood.

For HHV-6, immune control appeared delayed relative to the other HHVs, as continuous shedding and viral load were equivalent between children with primary and chronic infection but decreased in adult women. Yet, there was a wide range of shedding rates observed among both HIV-positive and -negative mothers in our cohort, and the median shedding rate exceeded 50%. As with the other HHVs, it is unclear if high shedding rates observed in certain mothers were due to re-infection or reactivation^19–21^.

EBV was unique among these viruses in that HIV infection was a clear risk factor for enhanced oral shedding rate and potentially higher viral load in mothers, implying that deficits in cell-associated mucosal immunity allow adults with HIV infection to control EBV as poorly as infants with primary infection. The clinicopathologic correlate of high oral EBV viral loads in people with HIV/AIDS is oral hairy leukoplakia^22^. Increased EBV shedding in the setting of HIV co-infection has been reported^23,24^. While CMV is associated with numerous HIV-associated opportunistic infections, HIV-positive and -negative mothers controlled CMV in the oral compartment equally effectively, and had considerably lower shedding rates than children. Similarly, the HSV shedding rate also was similar between HIV-positive and -negative mothers. These findings differ from other reports suggesting increased oral CMV and HSV shedding among adults with HIV infection^24^, but are consistent with the lack of difference observed in genital HSV-2 shedding due to HIV status in pesrons whose CD4 counts are preserved^25^. Of note, the HIV-positive women in our cohort had relatively high CD4 T cell counts^4^, which may influence control of oral shedding of these viruses^26^. We unfortunately did not collect maternal HIV viral load data in our study.

Our study has several limitations. First, swabs were collected weekly. While this should have no bearing on our determinations of shedding rate and quantity, which depend only on swab sample size, we may miss viral shedding episodes (defined as positive oral swabs followed by viral clearance in the oral mucosa), particularly but not limited to those that are less than a week in length.

Second, our cohorts were heterogeneous with respect to age and other demographic features and this may impact shedding kinetics. Our findings from mothers may or may not be generalizable to other populaions such as men who have sex with men. We nevertheless believe that our summary statistics provide a comprehensive ecologic overview of HHV shedding patterns in a sub-Saharan African setting.

Third, we were not able to perform typing on all swabs to distinguish HSV-1 from 2 or HHV-6A from 6B; however, the majority of oral shedding of these viruses is due to HSV-1 and HHV-6B^3,27,28^.

Fourth, the categorization of age and timing of infection was not precise for all analyses, and it was not feasible to study all household members (e.g., fathers). It was also not possible to determine at what point an infection changed from acute to chronic, and if all oral shedding was followed by systemic infection and subsequent seroconversion; as such, children with primary infection were categorized throughout follow-up. Some women may not have been infected with viruses that they did not shed, and for which serology was not performed. However, given the near universal rates of infection in sub-Saharan Africa with the HHVs studied^1,2,4,29^, this effect is likely negligible; indeed, most mothers shed HHV-6 and EBV in this cohort. Similarly, 16% of secondary children did not shed HHV-6 and were not included in the analysis but could theoretically have had chronic infection.

Finally, we can not exclude the possibility of a small number of false negative and positive swabs. Occasional error in swabbing technique may have allowed for false negative values or statistical noise. Based on the very clear patterns of shedding which emerge from our analysis, we believe these effects are minimal. Our previous demonstration of nearly continuous shedding during primry CMV infection with highly reproducible patterns of viral expansion, clearance and re-expansion across infants, clearly indicates that swabs were obtained in a consistent and rigorous fashion in all study participants^9,10^. Our cutoff of 3 DNA copies per reaction has been optimized for HSV infection but not for other HHVs^30^. Some low level positive results could theoretically represent environmental contamination rather than true viral replication.

In summary, we demonstrate the first kinetic picture of multiple primary viral infections in a prospective study cohort and compare shedding patterns of these viruses according to age and time of infection. We show that each HHV has its own shedding fingerprint, which in turn may have important implications for transmission dynamics and prevention strategies. Future studies are indicated to better understand the complex interplay between host and pathogen that results in highly specific shedding patterns.

## Methods

### Ethics statement

All methods were carried out in accordance with relevant international research and human subjects guidelines and regulations. All experimental protocols were approved by human subjects protection committees in Kampala, Uganda; Seattle, Washington; and Vancouver, Canada. Informed consent was obtained from all subjects.

### Study cohort and data collection

A cohort of pregnant women with at least one other child <7 years old (“secondary children”) in the home were enrolled in Kampala, Uganda as previously described^4^. Oral swabs were collected from all the children and their mothers weekly starting from the birth of the new infant. Blood was collected from mothers and secondary children at baseline and after one year, and every four months from infants. Human subjects review committees approved all study procedures. HIV serostatus was determined using the documentation in the medical record of the results of standard HIV testing per Ugandan national guidelines^31^.

### Laboratory assays

Swabs and plasma samples were tested by real-time quantitative (q)PCR for HSV-1 or 2, EBV, CMV, and HHV-6A or B using published methods^4,32–37^. The cutoffs for a positive test for swabs were three copies/reaction (~150 copies/mL of swab buffer) and one copy/reaction (50 copies/mL) for plasma. Typing was attempted for every sample with detectable HSV, and HHV-6 typing was performed for >1 sample from each subject with at least two positive results. Serology for CMV (IgM and IgG) and EBV (IgM and IgG against the viral capsid antigen, and IgG against nuclear antigen) was performed using ELISA kits (Wampole (Alere), Boucherville, Quebec), and for HSV-1 and HSV-2 using type-specific Western blot^38^. These serology tests were performed for most infants, but for only a subset of the secondary children, to supplement qPCR data when distinguishing chronic from primary infection. For the mothers, CMV and HSV but not EBV serology testing was done as above. Antibodies to HHV-6A or 6B were not measured.

### Definitions of infection

**Figure 6:**
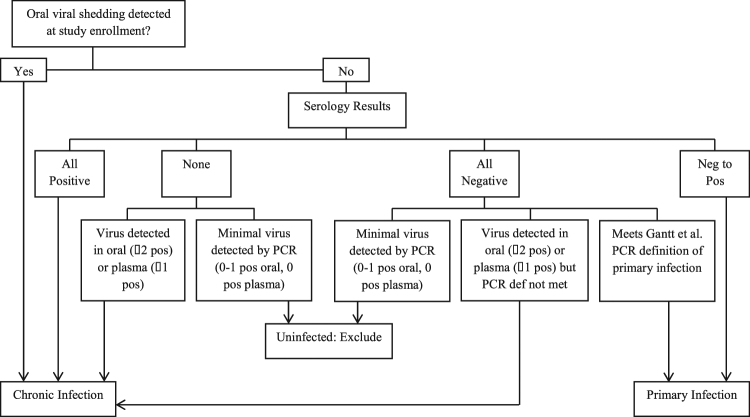
Infection classification scheme for secondary children in the study. Primary infection among secondary children was defined as those without viral DNA detected at the beginning of the study who either showed seroconversion or were seronegative and met the PCR-based definition of primary infection^31^. Children with minimal evidence of virus by PCR (<2 positive oral swabs, 0 positive plasma samples) and no positive serology were considered uninfected and excluded from shedding analysis for that virus. All remaining children were considered chronically infected.

For each virus, primary infection was defined by the onset of repeated detection of viral DNA detection in oral swabs and/or plasma of infants followed from birth^4^. Primary infection was defined for each virus in secondary children by seronegative status and no viral DNA detection in oral swabs at enrollment, followed by the onset of repeated detection of oral viral DNA detection in oral swabs and/or plasma [4] and/or seroconversion during the study. Secondary children with minimal evidence of virus by qPCR (<2 positive oral swabs, 0 positive plasma samples) and no positive serology were considered uninfected. All remaining secondary children were considered chronically infected (Fig. 6). For mothers, serology was used to confirm chronic infection with CMV and HSV. All mothers were assumed to have chronic infection with HHV-6 and EBV^2,4^.

### Statistical methods

For each virus, all participants were classified into one of four cohort groups for comparisons: 1) infants or children with primary infection; 2) children with chronic infection; 3) HIV-negative mothers; and 4) HIV-positive mothers. Children who were uninfected for a given HHV were excluded from analyses of that virus and children with 1 or fewer swabs

collected were excluded from all analyses. Mothers who were seronegative for CMV or HSV were excluded from analyses for those viruses, respectively. In the case of congenital infections, the entire family of the infant with congenital infection was excluded from analyses for that virus. Swabs collected prior to primary infection in infants and secondary children were excluded to restrict the analyses to time periods during infection. Given the small number of HIV-infected children (four), this group was not analyzed separately. A minority of oral HSV shedding was HSV-2 (nine primary infections of HSV-1 and one primary infection with HSV-2). However, because not all samples were typeable, HSV-1 and 2 data were analyzed together. Because detection of HHV-6A shedding was rare and was only observed in combination with HHV-6B^4^, HHV-6A and 6B were not analyzed separately.

Shedding rates were defined as the number of positive samples divided by the total number of samples. We compared oral shedding rates and viral loads in two ways: 1) within a given virus across cohort groups; and 2) within a given cohort group across viruses. Viral loads were only analyzed using positive swabs. Shedding rates and quantities were compared using generalized estimating equations (GEE) to account for correlation within participant, with an additional small-sample empirical correction of the variances^39,40^. Relative risks comparing shedding rates were estimated using a Poisson family with a log link^41^. Viral loads were compared using a Gaussian family. P-values were adjusted for multiple comparisons using the Holm step-down Bonferroni method^42^. Statistical models were programmed in the SAS programming language using the GLIMMIX procedure with the FIRORES empirical estimator^43^ and the STEPDOWN correction.

## Electronic supplementary material

Supplementary Information
