# Supplementary material for: Virus and host-specific differences in oral human herpesvirus shedding kinetics among Ugandan women and children
Source: Sci Rep. 2017 Oct 12;7:13105. doi: 10.1038/s41598-017-12994-0 (PMC5638921; doi:10.1038/s41598-017-12994-0)
Supplement: Supplementary file 1 — Supplementary Information [file 41598_2017_12994_MOESM1_ESM.pdf]

Supplementary Material for the manuscript:

"Virus and host-specific differences in oral human herpesvirus shedding kinetics among Ugandan women and children"

Laura Matrajt[a]*, Soren Gantt[a,b]*, Bryan T. Mayer[a], Elizabeth M. Krantz[a], Jackson Orem[c, d] Anna Wald[a,e,f,g], Lawrence Corey[a,e,g,h], Joshua T. Schiffer[a,e,h], Corey Casper[a,e,f,h,I,j]

[a]Fred Hutchinson Cancer Research Center, Seattle, WA, USA; [b]Department of Pediatrics, Division of Infectious Diseases, University of British Columbia, Vancouver, BC, CANADA; [c]Uganda Cancer Institute, Makerere University, Kampala, UGANDA; [d]College of Health Sciences, Makerere University, Kampala, UGANDA; [e]Department of Medicine, University of Washington, Seattle, WA 98195, USA; [f]Department of Epidemiology, University of Washington, Seattle, WA, USA; [g]Department of Laboratory Medicine, University of Washington, Seattle, WA, USA; [h]Clinical Research Division, Fred Hutchinson Cancer Research Center, Seattle, WA, USA; [i]Public Health Science Division, Fred Hutchinson Cancer Research Center, Seattle, WA, USA; [j]Department of Global Health, University of Washington, Seattle, WA, USA

*These authors contributed equally to this work.

Figure S1: **Oral HSV shedding patterns within families.** Each panel depicts the oral shedding of HSV in a single household. No HSV was detected within family 31 during the period of the study. Breaks in the lines indicate gaps between two samples of over 15 days.

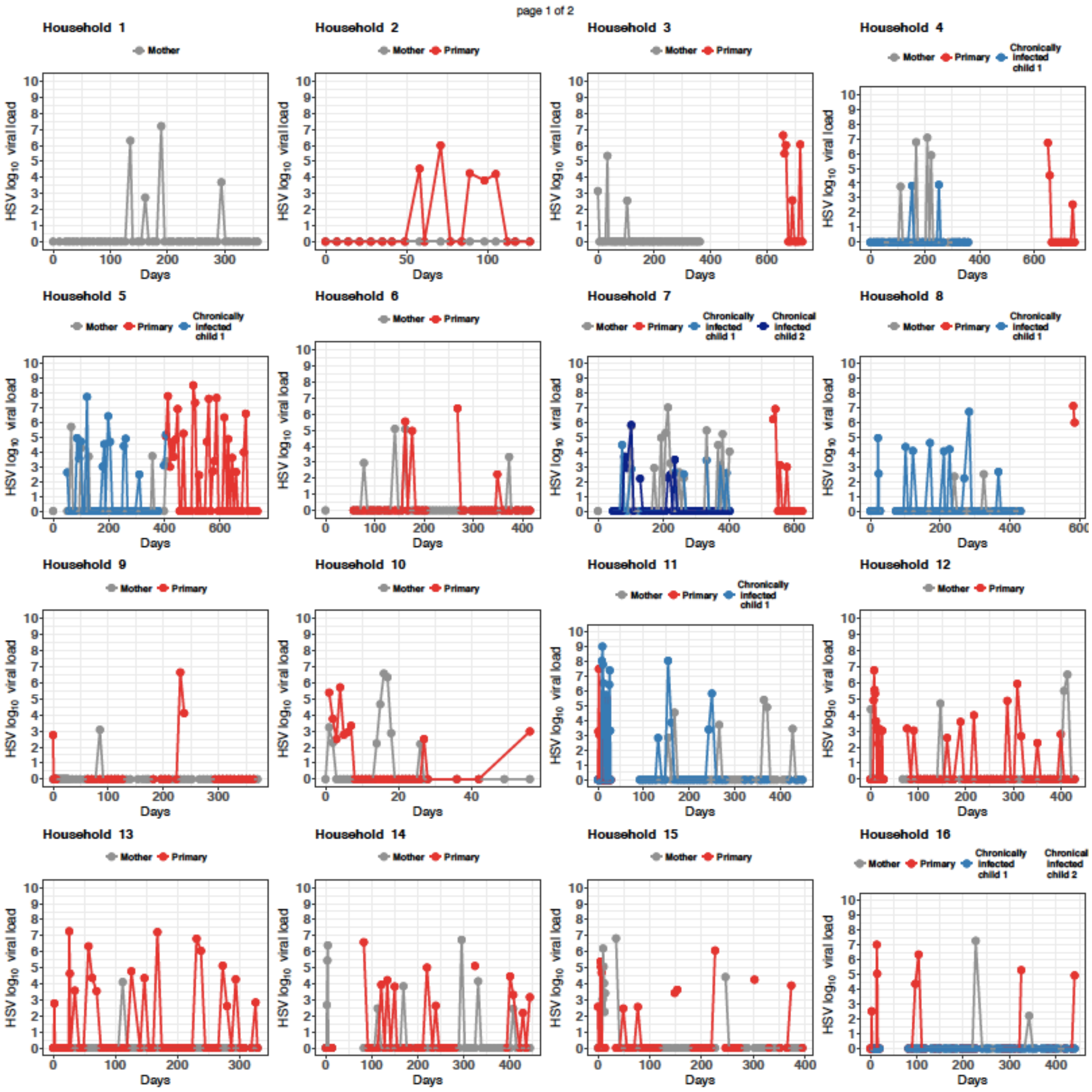

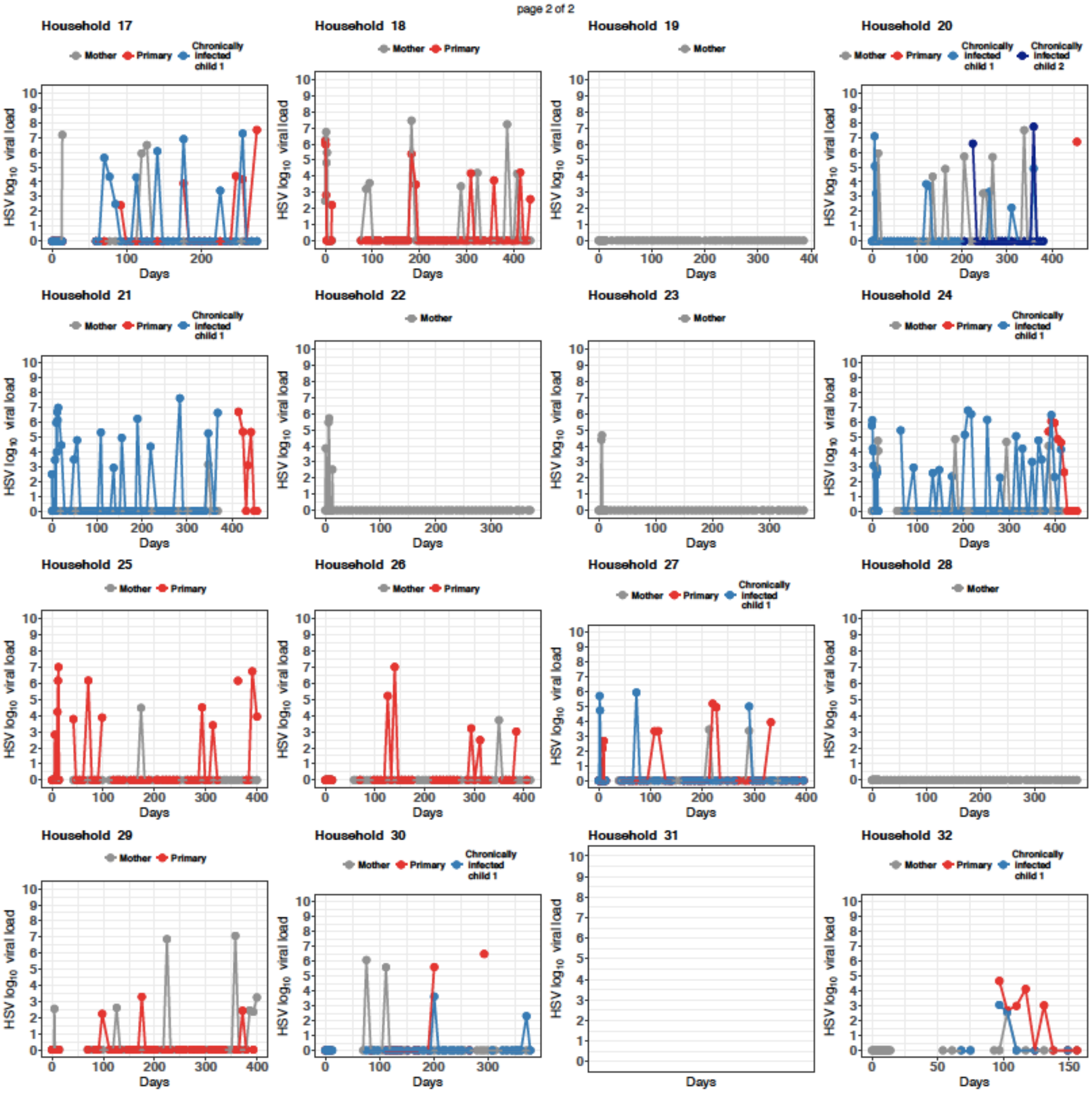
Household 17
Household 18
Household 19
Household 20
Household 21
Household 22
Household 23
Household 24
Household 25
Household 26
Household 27
Household 28
Household 29
Household 30
Household 31
Household 32
Mother
Primary
Chronically infected child 1
Chronically infected child 2
HSV $\log_{10}$ viral load
Days

Figure S2: **Oral EBV shedding patterns within families.** Each panel depicts the oral shedding of EBV in a single household. Breaks in the lines indicate gaps between two samples of over 15 days.

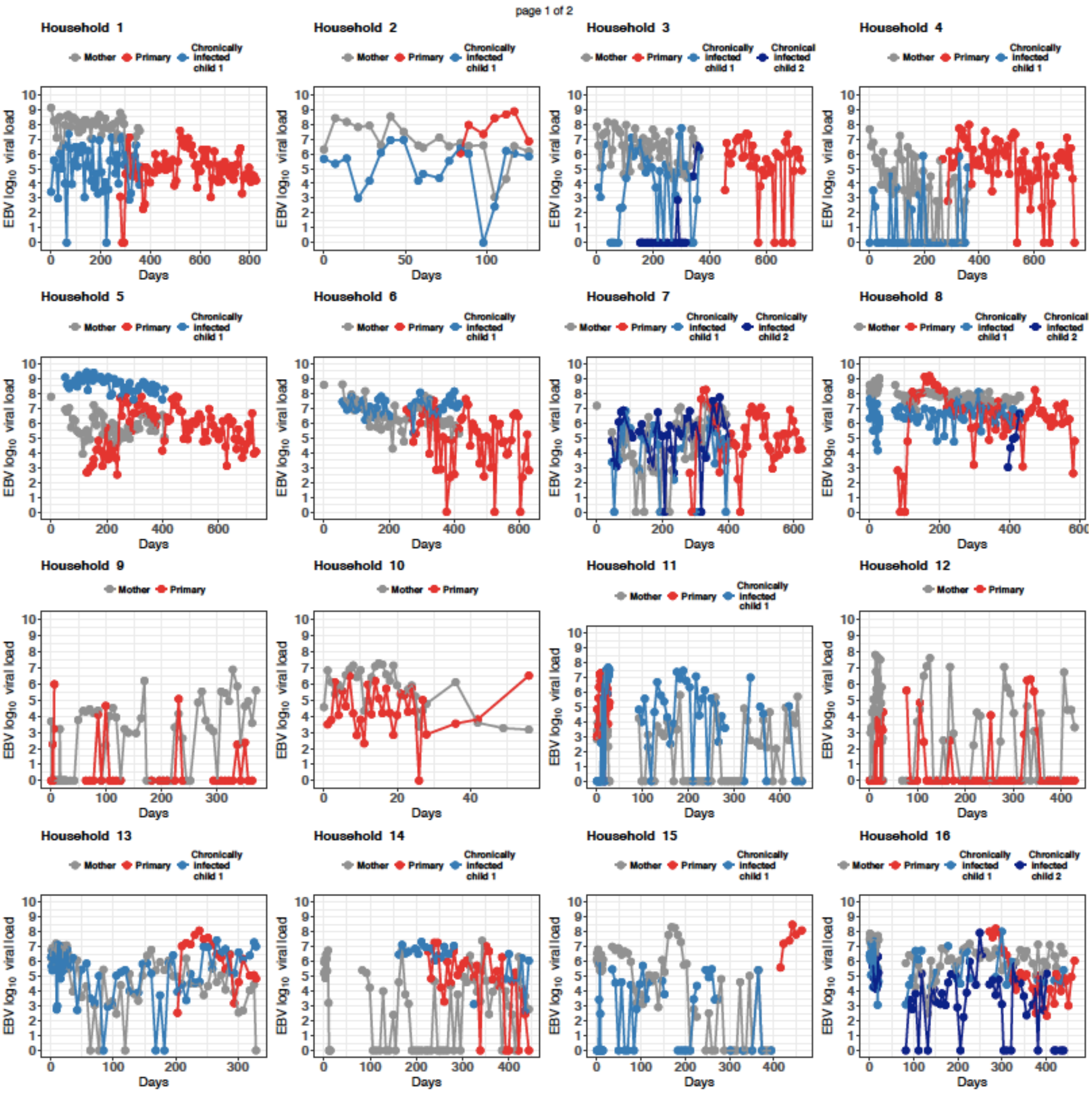

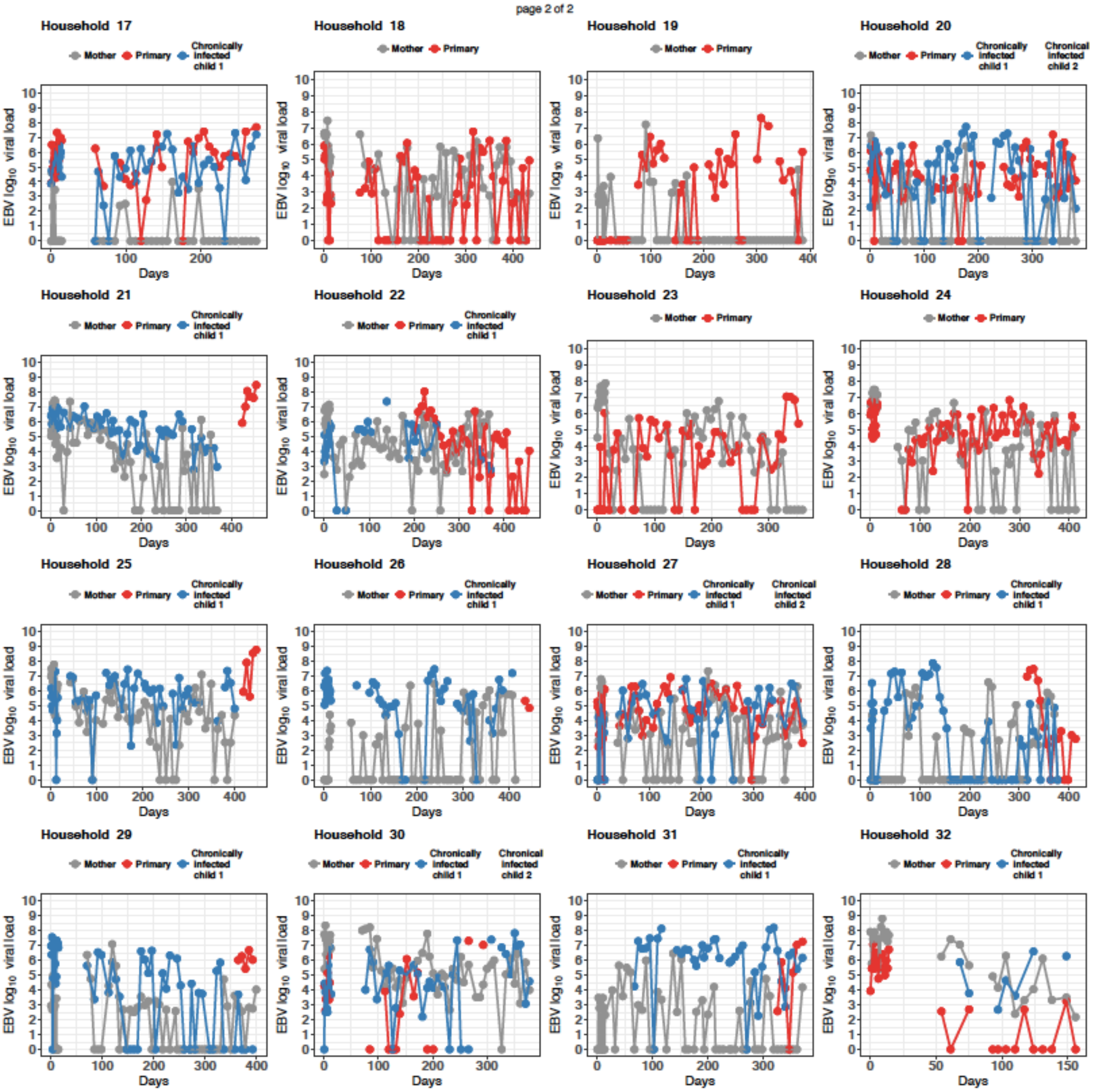
Household 17
Mother Primary Chronically infected child 1
Household 18
Mother Primary
Household 19
Mother Primary
Household 20
Mother Primary Chronically infected child 1 Chronical infected child 2
Household 21
Mother Primary Chronically infected child 1
Household 22
Mother Primary Chronically infected child 1
Household 23
Mother Primary
Household 24
Mother Primary
Household 25
Mother Primary Chronically infected child 1
Household 26
Mother Primary Chronically infected child 1
Household 27
Mother Primary Chronically infected child 1 Chronical infected child 2
Household 28
Mother Primary Chronically infected child 1
Household 29
Mother Primary Chronically infected child 1
Household 30
Mother Primary Chronically infected child 1 Chronical infected child 2
Household 31
Mother Primary Chronically infected child 1
Household 32
Mother Primary Chronically infected child 1
EBV log10 viral load
Days

Figure S3: **Oral CMV shedding patterns within families.** Each panel depicts the oral shedding of CMV in a single household. No CMV was detected within families 2 and 8 during the period of the study. Breaks in the lines indicate gaps between two samples of over 15 days.

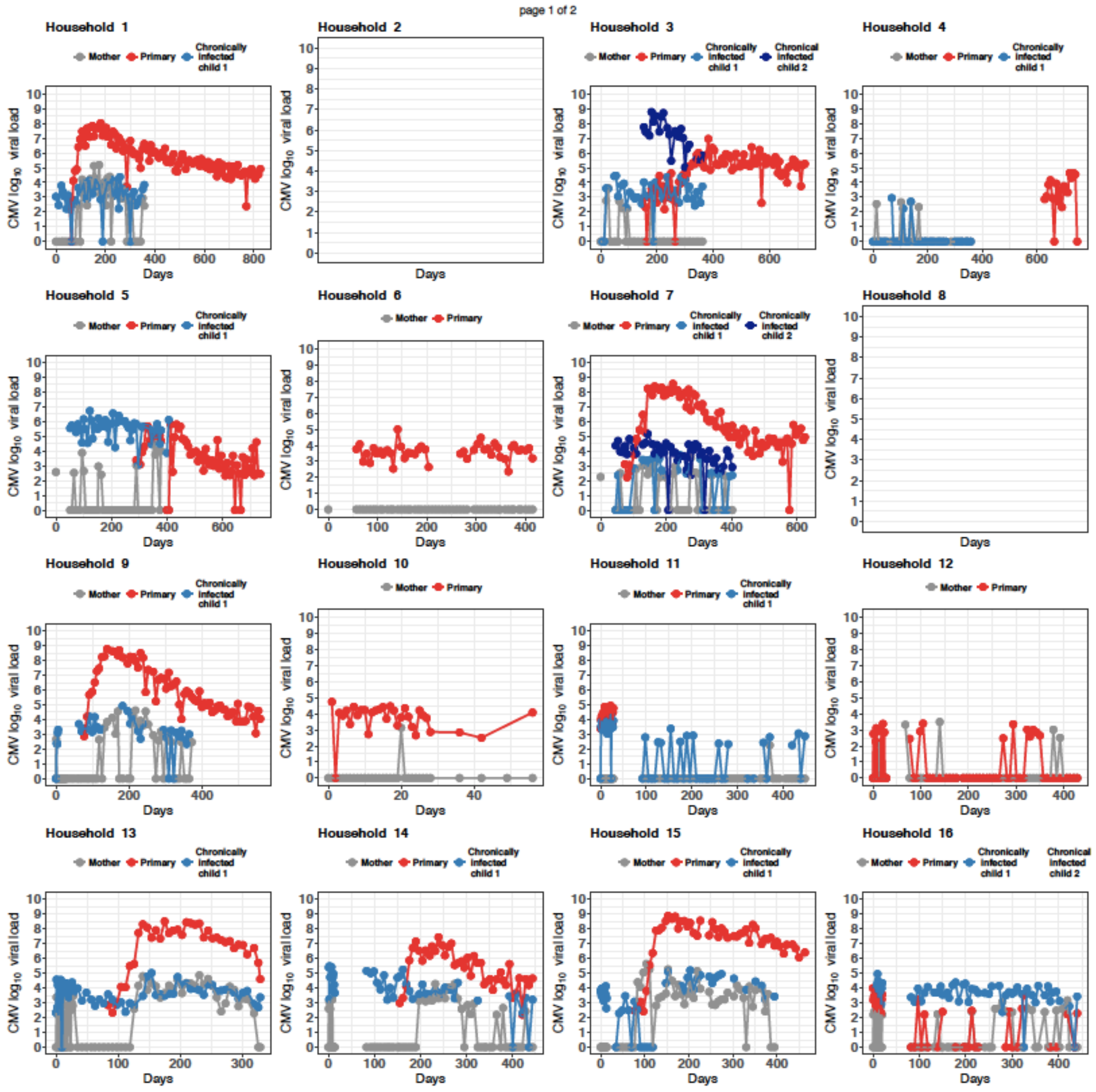

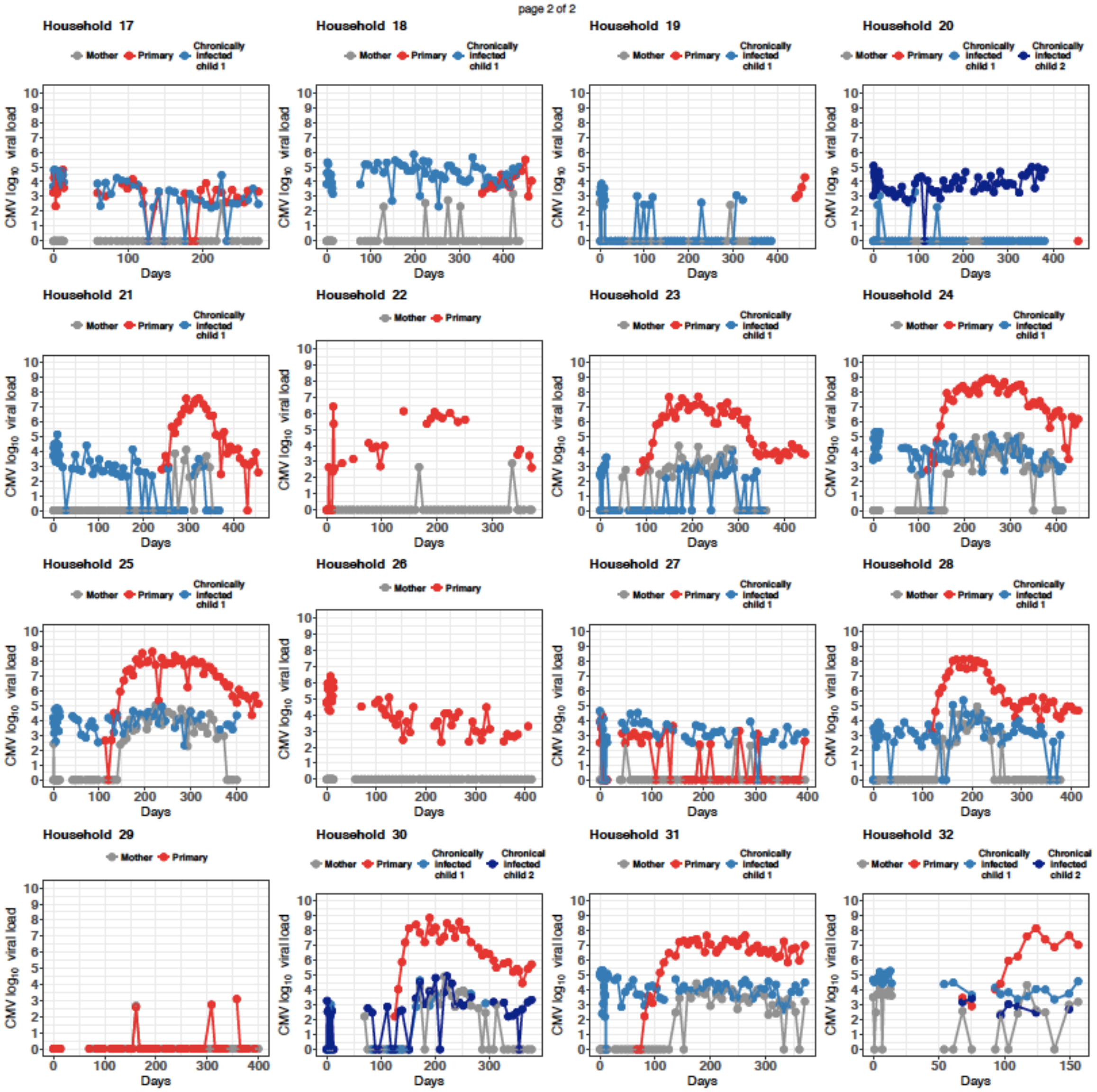
Household 17
Mother
Primary
Chronically infected child 1
CMV log10 viral load
Days
Household 18
Household 19
Household 20
Chronically infected child 2
Household 21
Household 22
Household 23
Household 24
Household 25
Household 26
Household 27
Household 28
Household 29
Household 30
Chronical infected child 2
Household 31
Household 32

Figure S4: **Oral HHV-6 shedding patterns within families.** Each panel depicts the oral shedding of HHV-6 in a single household. Breaks in the lines indicate gaps between two samples of over 15 days.

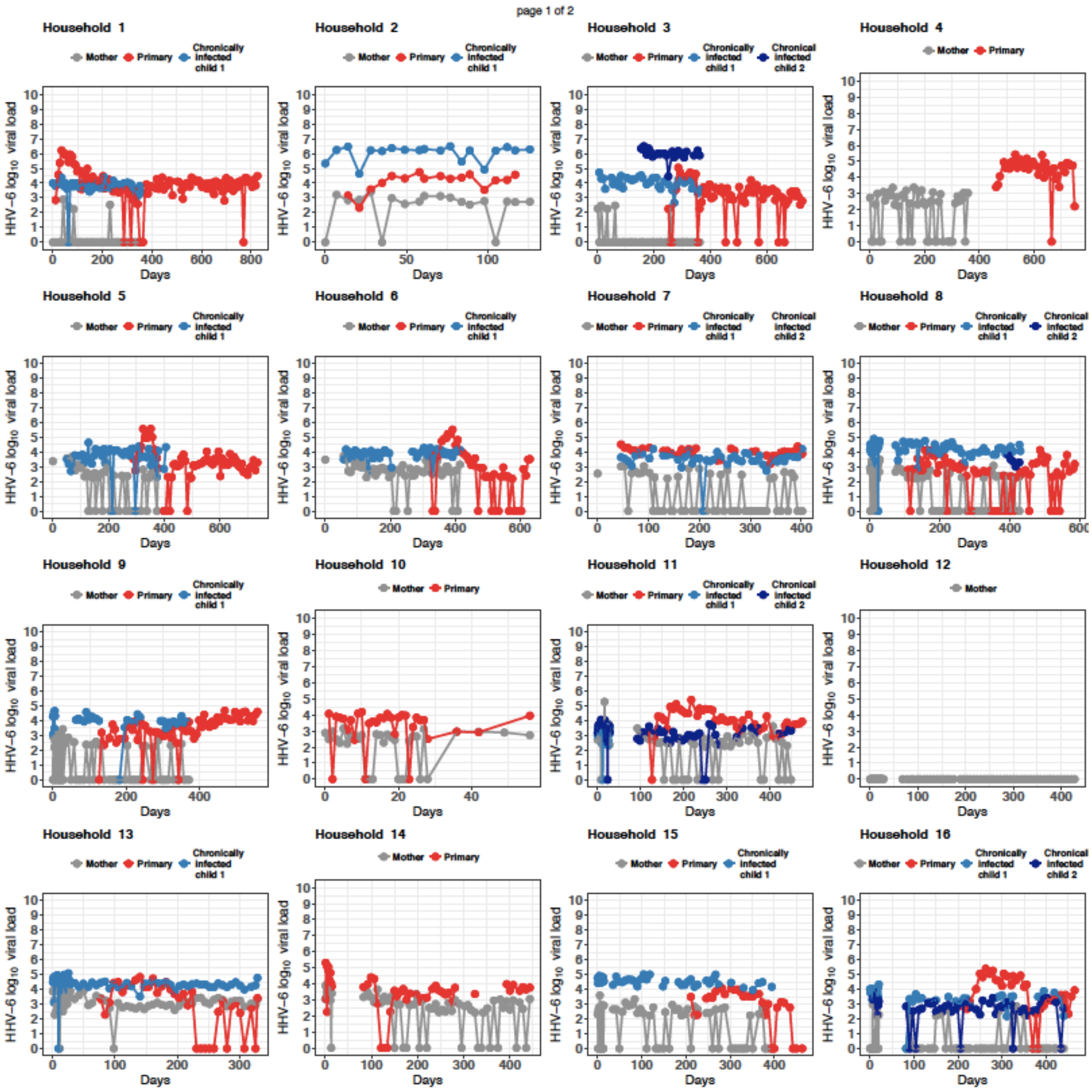

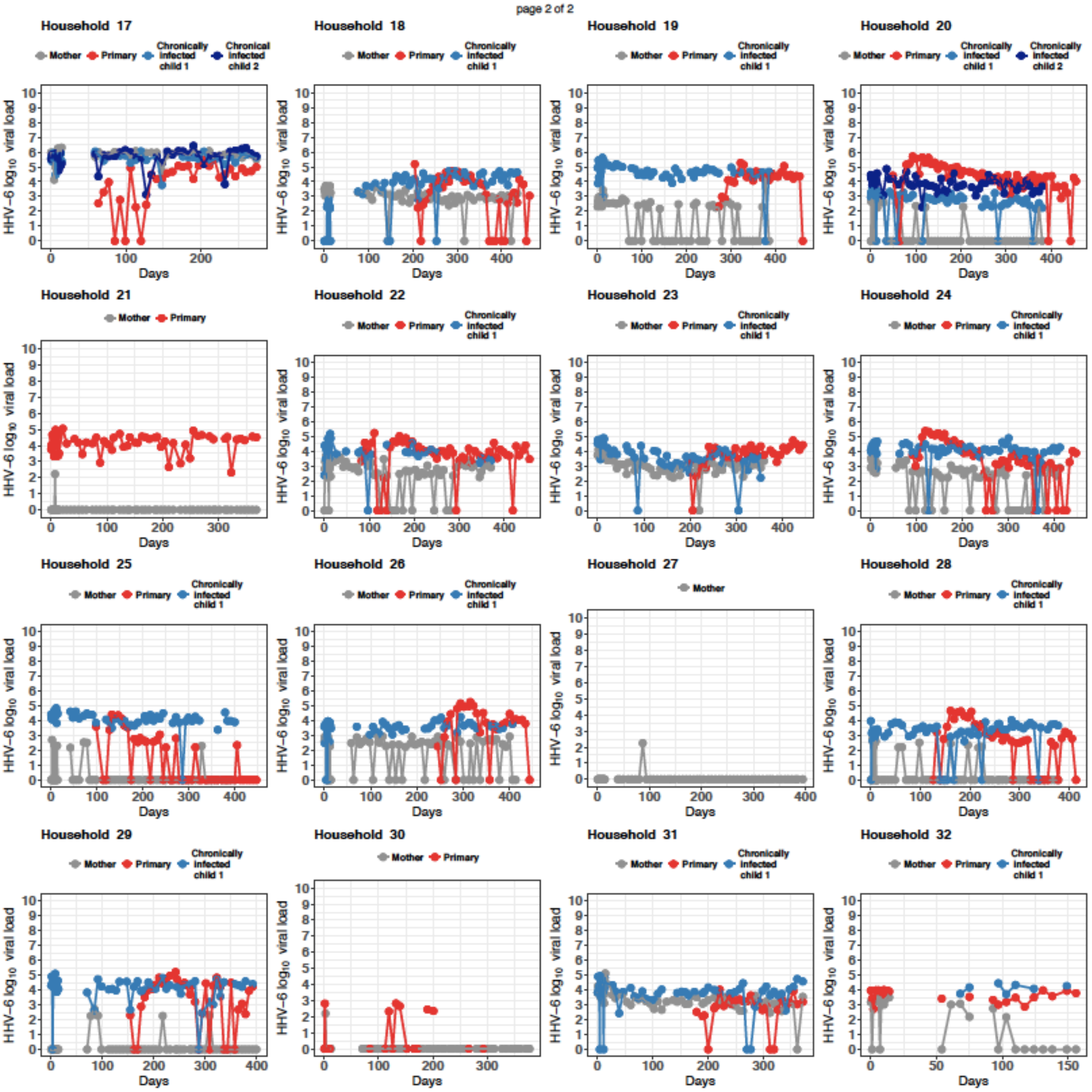
Household 17
Mother
Primary
Chronically infected child 1
Chronical infected child 2
Household 18
Household 19
Household 20
Household 21
Household 22
Household 23
Household 24
Household 25
Household 26
Household 27
Household 28
Household 29
Household 30
Household 31
Household 32
HHV-6 log10 viral load
Days

Figure S5: **Shedding patterns of children with primary infections with multiple human herpesviruses.** Beginning at the time of primary infection, shown is the quantity of viral DNA detected in oral swabs collected weekly from the time of birth (Day 0). Each panel shows data from one primary-infected child.

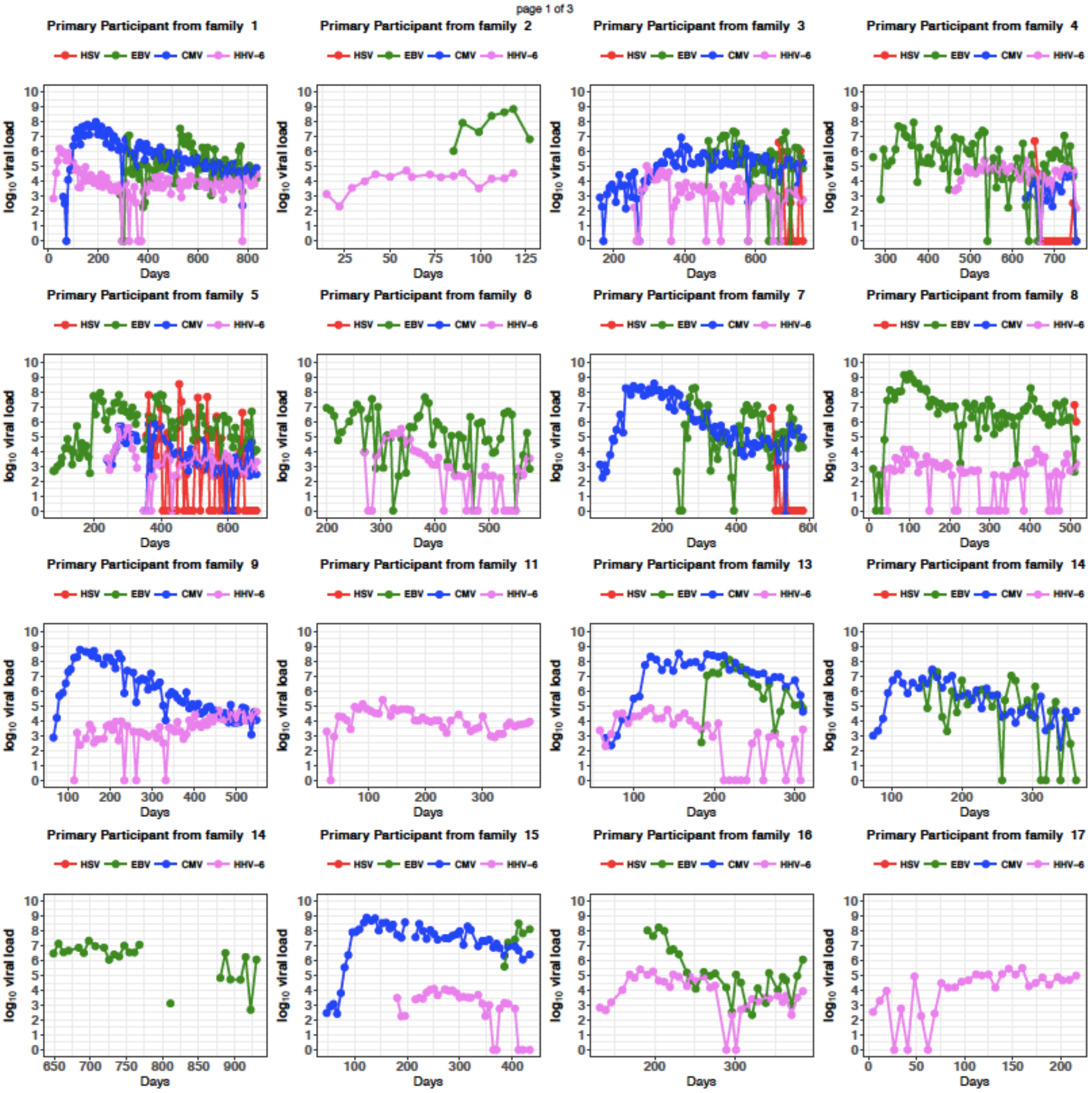

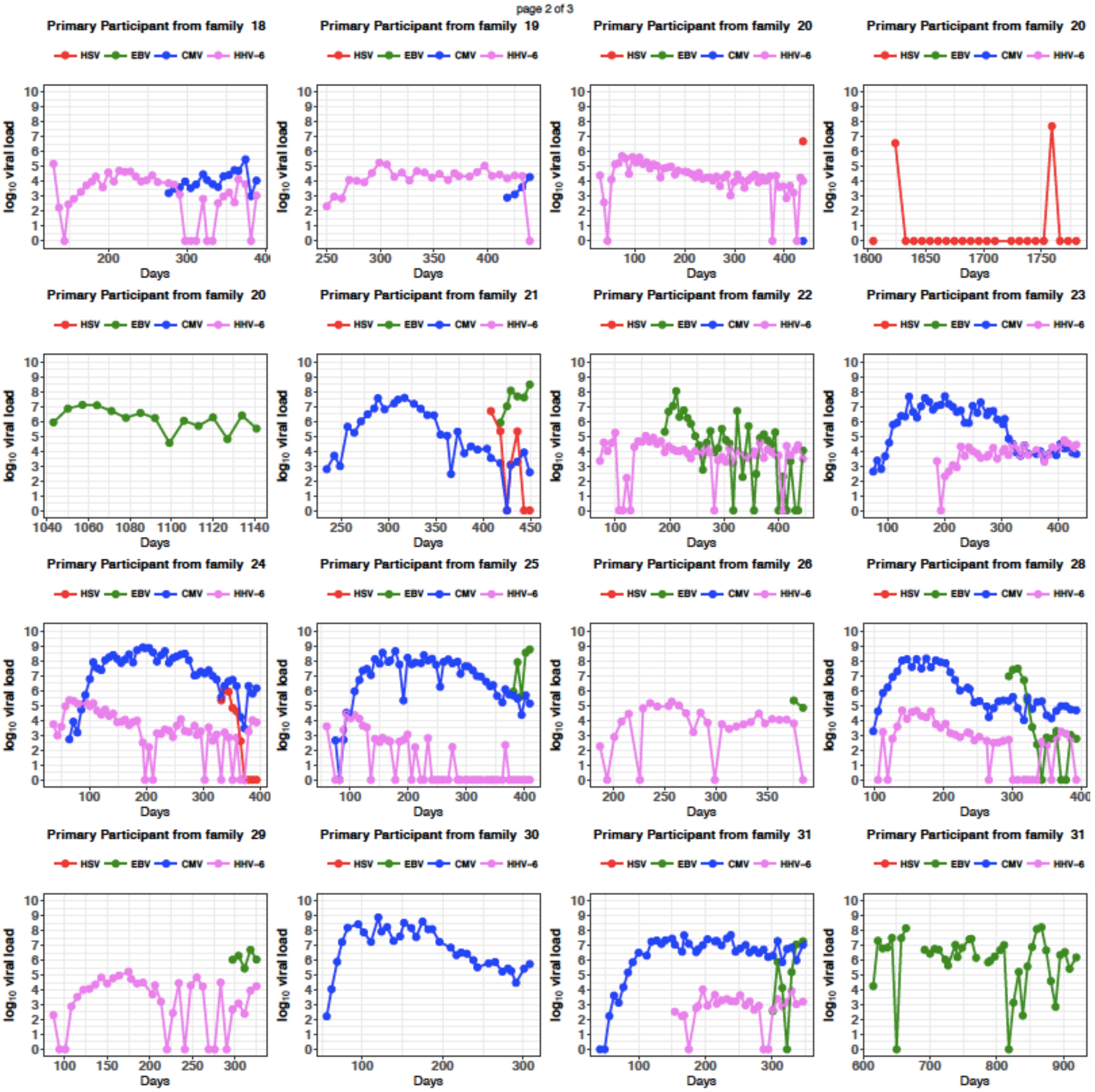
Primary Participant from family 18
Primary Participant from family 19
Primary Participant from family 20
Primary Participant from family 20
Primary Participant from family 20
Primary Participant from family 21
Primary Participant from family 22
Primary Participant from family 23
Primary Participant from family 24
Primary Participant from family 25
Primary Participant from family 26
Primary Participant from family 28
Primary Participant from family 29
Primary Participant from family 30
Primary Participant from family 31
Primary Participant from family 31
HSV
EBV
CMV
HHV-6
log10 viral load
Days

Figure S6: Shedding probability for each virus compared between cohort groups and within each cohort group compared between different viruses. Top panels **(A)** show comparisons between subject groups for each virus, while comparisons between viruses for each subject group are show in bottom panels **(B)**. Each dot represents the relative risk comparing the group to the left with the group below; dot size denotes the level of the effect and the fill color denotes statistical significance. For example, for the first panel in **A**, individuals in the primary infection group are 3 to 5 times more likely to shed HSV than chronically infected children, and over 5 times more likely to shed HSV compared to mothers, regardless of their HIV status. All of these differences are statistically significant.

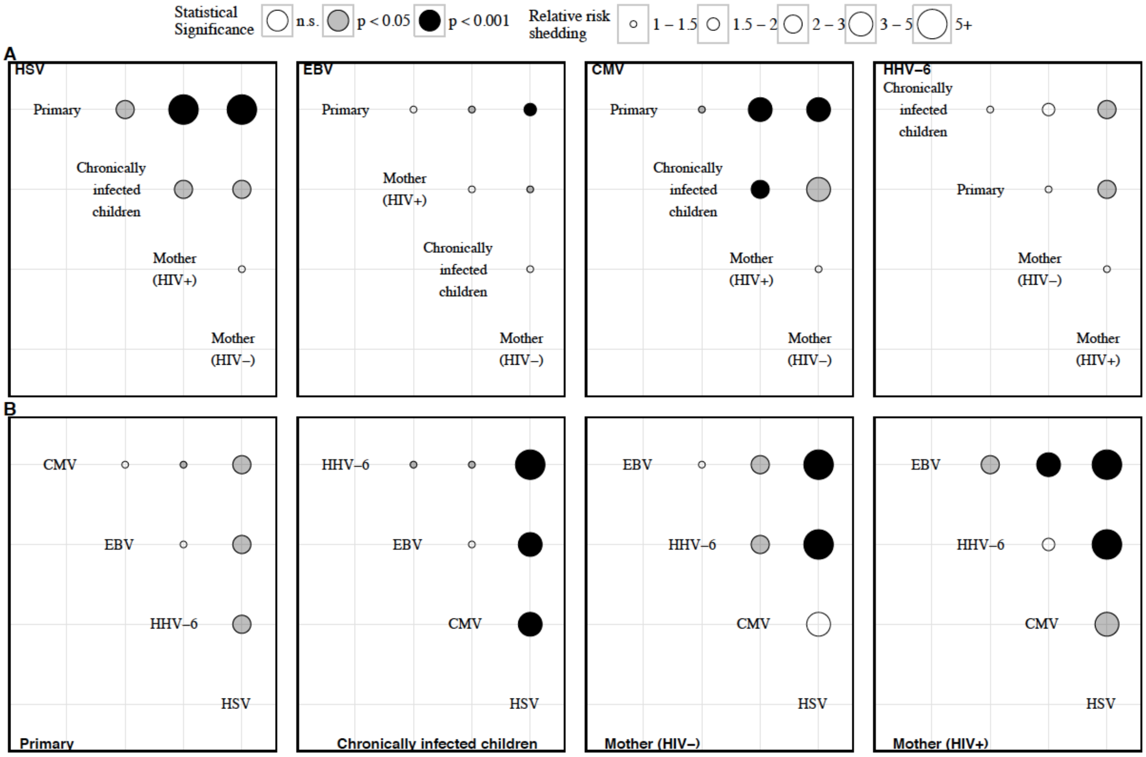

Figure S7: Difference in mean shedding quantity compared for each virus between cohort groups and within each cohort group between different viruses. Mean values were calculated for positive samples only. Top panels **(A)** show comparisons between subject groups for each virus, while comparisons between viruses for each subject group are show in bottom panels **(B)**. Each dot represents the mean shedding difference between the group to the left with the group below; dot size denotes the magnitude of this difference (in $\log_{10}$ copies/swab) and the fill denotes statistical significance. For example, for the middle top panel in **(A)**, the mean shedding difference of CMV between individuals in the primary infection group and chronically infected children is between 1 and 2 $\log_{10}$ copies/swab, this difference increases to 2 to 3 $\log_{10}$ copies/swab when comparing the primary infection group to the mothers, regardless of their HIV status (p-value< 0.001).

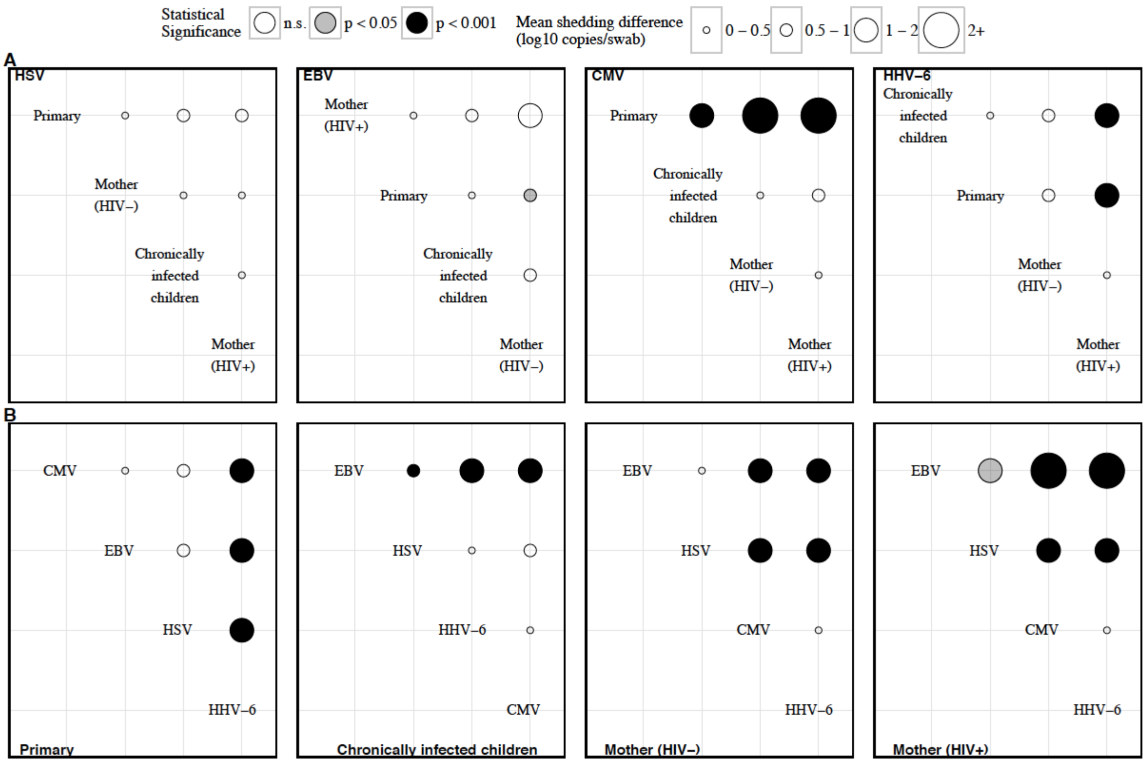

Table S1: Relative risk of shedding probabilities between viruses and cohorts.

Analysis	Ref. virus	Comp. virus	Ref. group	Comp. group	Relative risk	Raw p value	Adj. p value
within virus	HHV6	HHV6	M	H	0.735	0.241	1.000
within virus	HHV6	HHV6	P	H	0.496	0.001	0.014
within virus	HHV6	HHV6	C	H	0.546	0.004	0.083
within virus	HHV6	HHV6	P	M	0.674	0.029	0.410
within virus	HHV6	HHV6	C	M	0.742	0.122	1.000
within virus	HHV6	HHV6	C	P	1.101	0.245	1.000
within virus	EBV	EBV	M	H	1.428	0.002	0.046
within virus	EBV	EBV	P	H	0.911	0.174	1.000
within virus	EBV	EBV	C	H	1.079	0.359	1.000
within virus	EBV	EBV	P	M	0.638	0.000	0.000
within virus	EBV	EBV	C	M	0.755	0.008	0.141
within virus	EBV	EBV	C	P	1.184	0.001	0.023
within virus	CMV	CMV	M	H	1.180	0.637	1.000
within virus	CMV	CMV	P	H	0.265	0.000	0.000
within virus	CMV	CMV	C	H	0.340	0.000	0.000
within virus	CMV	CMV	P	M	0.224	0.000	0.000
within virus	CMV	CMV	C	M	0.288	0.000	0.001
within virus	CMV	CMV	C	P	1.283	0.000	0.005
within virus	HSV	HSV	M	H	1.215	0.573	1.000
within virus	HSV	HSV	P	H	0.163	0.000	0.000
within virus	HSV	HSV	C	H	0.553	0.024	0.353
within virus	HSV	HSV	P	M	0.134	0.000	0.000
within virus	HSV	HSV	C	M	0.455	0.010	0.171
within virus	HSV	HSV	C	P	3.384	0.000	0.000
within group	EBV	CMV	P	P	1.037	0.037	0.486
within group	HHV6	CMV	P	P	1.135	0.001	0.014
within group	HSV	CMV	P	P	2.212	0.000	0.000
within group	HHV6	EBV	P	P	1.095	0.013	0.210
within group	HSV	EBV	P	P	2.133	0.000	0.000
within group	HSV	HHV6	P	P	1.948	0.000	0.000
within group	EBV	CMV	C	C	0.957	0.527	1.000
within group	HHV6	CMV	C	C	0.974	0.765	1.000
within group	HSV	CMV	C	C	5.831	0.000	0.000
within group	HHV6	EBV	C	C	1.018	0.807	1.000
within group	HSV	EBV	C	C	6.095	0.000	0.000
within group	HSV	HHV6	C	C	5.985	0.000	0.000
within group	EBV	CMV	M	M	0.364	0.000	0.005
within group	HHV6	CMV	M	M	0.378	0.001	0.014
within group	HSV	CMV	M	M	3.687	0.003	0.069
within group	HHV6	EBV	M	M	1.036	0.853	1.000
within group	HSV	EBV	M	M	10.120	0.000	0.000
within group	HSV	HHV6	M	M	9.767	0.000	0.000
within group	EBV	CMV	H	H	0.301	0.000	0.000
within group	HHV6	CMV	H	H	0.606	0.139	1.000
within group	HSV	CMV	H	H	3.582	0.000	0.007
within group	HHV6	EBV	H	H	2.013	0.001	0.017
within group	HSV	EBV	H	H	11.896	0.000	0.000
within group	HSV	HHV6	H	H	5.910	0.000	0.000

Abbreviations: Ref. = reference, Comp. = comparator, P= primary children, C= chronically-infected children, M=HIV-negative mothers, H=HIV-positive mothers.

Table S2: Differences in shedding magnitude between viruses and cohorts.

Ref. virus	Comp. virus	Ref. group	Comp. group	Mean diff. (log_{10} virus copies)	Adj. p value	95% confidence interval
CMV	CMV	M	H	-0.177	1.000	-0.854, 0.500
CMV	CMV	P	H	-2.506	0.000	-3.529, -1.484
CMV	CMV	C	H	-0.569	0.200	-1.260, 0.122
EBV	CMV	H	H	-2.638	0.000	-3.995, -1.280
EBV	CMV	M	H	-1.510	0.000	-2.303, -0.717
EBV	CMV	P	H	-2.439	0.000	-3.269, -1.609
EBV	CMV	C	H	-2.086	0.000	-2.884, -1.289
HHV6	CMV	H	H	0.451	0.297	-0.125, 1.028
HHV6	CMV	M	H	0.055	1.000	-0.995, 1.104
HHV6	CMV	P	H	-0.560	0.144	-1.217, 0.096
HHV6	CMV	C	H	-0.724	0.013	-1.411, -0.036
HHV8	CMV	H	H	-1.195	0.000	-1.885, -0.505
HHV8	CMV	M	H	-0.713	1.000	-2.573, 1.148
HHV8	CMV	C	H	-1.450	0.000	-2.433, -0.467
HSV	CMV	H	H	-1.054	0.001	-1.898, -0.210
HSV	CMV	M	H	-1.358	0.000	-2.311, -0.405
HSV	CMV	P	H	-1.774	0.000	-2.732, -0.816
HSV	CMV	C	H	-1.073	0.000	-1.822, -0.324
CMV	CMV	P	M	-2.329	0.000	-3.263, -1.396
CMV	CMV	C	M	-0.392	0.551	-0.944, 0.160
EBV	CMV	H	M	-2.460	0.000	-3.750, -1.171
EBV	CMV	M	M	-1.333	0.000	-1.821, -0.845
EBV	CMV	P	M	-2.262	0.000	-2.980, -1.543
EBV	CMV	C	M	-1.909	0.000	-2.590, -1.229
HHV6	CMV	H	M	0.628	0.000	0.208, 1.049
HHV6	CMV	M	M	0.232	1.000	-0.777, 1.241
HHV6	CMV	P	M	-0.383	0.389	-0.891, 0.124
HHV6	CMV	C	M	-0.547	0.026	-1.094, 0.001
HHV8	CMV	H	M	-1.018	0.000	-1.651, -0.385
HHV8	CMV	M	M	-0.536	1.000	-2.110, 1.039
HHV8	CMV	C	M	-1.273	0.000	-2.164, -0.382
HSV	CMV	H	M	-0.877	0.001	-1.592, -0.162
HSV	CMV	M	M	-1.181	0.000	-2.105, -0.256
HSV	CMV	P	M	-1.597	0.000	-2.460, -0.734
HSV	CMV	C	M	-0.896	0.000	-1.519, -0.273
CMV	CMV	C	P	1.938	0.000	0.993, 2.882
EBV	CMV	H	P	-0.131	1.000	-1.631, 1.369
EBV	CMV	M	P	0.996	0.035	-0.025, 2.018
EBV	CMV	P	P	0.068	1.000	-0.909, 1.045
EBV	CMV	C	P	0.420	1.000	-0.605, 1.445
HHV6	CMV	H	P	2.958	0.000	2.084, 3.832
HHV6	CMV	M	P	2.561	0.000	1.330, 3.792
HHV6	CMV	P	P	1.946	0.000	1.039, 2.853
HHV6	CMV	C	P	1.783	0.000	0.841, 2.725
HHV8	CMV	H	P	1.311	0.000	0.318, 2.305
HHV8	CMV	M	P	1.794	0.075	-0.175, 3.762
HHV8	CMV	C	P	1.056	0.086	-0.119, 2.231
HSV	CMV	H	P	1.452	0.000	0.404, 2.500

HSV	CMV	M	P	1.149	0.026	-0.001, 2.299
HSV	CMV	P	P	0.732	1.000	-0.631, 2.095
HSV	CMV	C	P	1.433	0.000	0.446, 2.421
EBV	CMV	H	C	-2.069	0.000	-3.366, -0.772
EBV	CMV	M	C	-0.941	0.000	-1.631, -0.252
EBV	CMV	P	C	-1.870	0.000	-2.594, -1.146
EBV	CMV	C	C	-1.518	0.000	-2.080, -0.956
HHV6	CMV	H	C	1.020	0.000	0.577, 1.463
HHV6	CMV	M	C	0.623	1.000	-0.350, 1.597
HHV6	CMV	P	C	0.008	1.000	-0.518, 0.535
HHV6	CMV	C	C	-0.155	1.000	-0.682, 0.373
HHV8	CMV	H	C	-0.626	0.038	-1.274, 0.022
HHV8	CMV	M	C	-0.144	1.000	-1.963, 1.675
HHV8	CMV	C	C	-0.881	0.035	-1.786, 0.023
HSV	CMV	H	C	-0.486	0.830	-1.214, 0.243
HSV	CMV	M	C	-0.789	0.077	-1.658, 0.080
HSV	CMV	P	C	-1.205	0.000	-2.080, -0.331
HSV	CMV	C	C	-0.504	0.402	-1.176, 0.168
EBV	EBV	M	H	1.127	0.180	-0.227, 2.482
EBV	EBV	P	H	0.199	1.000	-1.178, 1.575
EBV	EBV	C	H	0.551	1.000	-0.806, 1.908
HHV6	EBV	H	H	3.089	0.000	1.783, 4.395
HHV6	EBV	M	H	2.692	0.000	1.174, 4.211
HHV6	EBV	P	H	2.077	0.000	0.798, 3.356
HHV6	EBV	C	H	1.914	0.000	0.619, 3.209
HHV8	EBV	H	H	1.443	0.000	0.454, 2.431
HHV8	EBV	M	H	1.925	0.093	-0.235, 4.085
HHV8	EBV	C	H	1.187	0.236	-0.286, 2.661
HSV	EBV	H	H	1.583	0.012	0.087, 3.079
HSV	EBV	M	H	1.280	0.105	-0.174, 2.733
HSV	EBV	P	H	0.863	1.000	-0.594, 2.320
HSV	EBV	C	H	1.565	0.002	0.236, 2.893
EBV	EBV	P	M	-0.929	0.005	-1.758, -0.100
EBV	EBV	C	M	-0.576	0.483	-1.372, 0.219
HHV6	EBV	H	M	1.961	0.000	1.372, 2.551
HHV6	EBV	M	M	1.565	0.000	0.463, 2.666
HHV6	EBV	P	M	0.950	0.000	0.295, 1.604
HHV6	EBV	C	M	0.786	0.003	0.101, 1.472
HHV8	EBV	H	M	0.315	1.000	-0.441, 1.071
HHV8	EBV	M	M	0.797	1.000	-0.894, 2.489
HHV8	EBV	C	M	0.060	1.000	-0.922, 1.042
HSV	EBV	H	M	0.456	1.000	-0.370, 1.282
HSV	EBV	M	M	0.152	1.000	-0.766, 1.070
HSV	EBV	P	M	-0.264	1.000	-1.221, 0.693
HSV	EBV	C	M	0.437	1.000	-0.310, 1.185
EBV	EBV	C	P	0.352	1.000	-0.481, 1.185
HHV6	EBV	H	P	2.890	0.000	2.251, 3.529
HHV6	EBV	M	P	2.493	0.000	1.417, 3.570
HHV6	EBV	P	P	1.879	0.000	1.046, 2.711
HHV6	EBV	C	P	1.715	0.000	0.978, 2.452
HHV8	EBV	H	P	1.244	0.000	0.449, 2.038
HHV8	EBV	M	P	1.726	0.068	-0.150, 3.602
HHV8	EBV	C	P	0.989	0.035	-0.024, 2.001
HSV	EBV	H	P	1.385	0.000	0.523, 2.246

HSV	EBV	M	P	1.081	0.007	0.098, 2.064
HSV	EBV	P	P	0.665	0.453	-0.238, 1.567
HSV	EBV	C	P	1.366	0.000	0.572, 2.159
HHV6	EBV	H	C	2.538	0.000	1.943, 3.133
HHV6	EBV	M	C	2.141	0.000	1.090, 3.193
HHV6	EBV	P	C	1.526	0.000	0.866, 2.186
HHV6	EBV	C	C	1.363	0.000	0.709, 2.017
HHV8	EBV	H	C	0.891	0.002	0.131, 1.652
HHV8	EBV	M	C	1.374	0.453	-0.488, 3.236
HHV8	EBV	C	C	0.636	1.000	-0.375, 1.648
HSV	EBV	H	C	1.032	0.001	0.202, 1.862
HSV	EBV	M	C	0.729	0.368	-0.227, 1.684
HSV	EBV	P	C	0.312	1.000	-0.648, 1.273
HSV	EBV	C	C	1.013	0.000	0.348, 1.679
HHV6	HHV6	M	H	-0.397	1.000	-1.302, 0.508
HHV6	HHV6	P	H	-1.012	0.000	-1.398, -0.625
HHV6	HHV6	C	H	-1.175	0.000	-1.613, -0.737
HHV8	HHV6	H	H	-1.646	0.000	-2.202, -1.091
HHV8	HHV6	M	H	-1.164	0.932	-2.947, 0.619
HHV8	HHV6	C	H	-1.902	0.000	-2.729, -1.074
HSV	HHV6	H	H	-1.506	0.000	-2.129, -0.882
HSV	HHV6	M	H	-1.809	0.000	-2.601, -1.017
HSV	HHV6	P	H	-2.226	0.000	-3.023, -1.428
HSV	HHV6	C	H	-1.524	0.000	-2.053, -0.995
HHV6	HHV6	P	M	-0.615	0.960	-1.564, 0.334
HHV6	HHV6	C	M	-0.778	0.245	-1.749, 0.193
HHV8	HHV6	H	M	-1.250	0.001	-2.271, -0.228
HHV8	HHV6	M	M	-0.767	1.000	-2.817, 1.283
HHV8	HHV6	C	M	-1.505	0.001	-2.703, -0.306
HSV	HHV6	H	M	-1.109	0.017	-2.183 -0.034
HSV	HHV6	M	M	-1.412	0.000	-2.221, -0.604
HSV	HHV6	P	M	-1.829	0.000	-3.007, -0.651
HSV	HHV6	C	M	-1.128	0.006	-2.143, -0.112
HHV6	HHV6	C	P	-0.163	1.000	-0.686, 0.359
HHV8	HHV6	H	P	-0.635	0.015	-1.246, -0.024
HHV8	HHV6	M	P	-0.152	1.000	-1.958, 1.653
HHV8	HHV6	C	P	-0.890	0.021	-1.765, -0.014
HSV	HHV6	H	P	-0.494	0.551	-1.190, 0.202
HSV	HHV6	M	P	-0.798	0.048	-1.639, 0.044
HSV	HHV6	P	P	-1.214	0.000	-2.149, -0.279
HSV	HHV6	C	P	-0.513	0.144	-1.114, 0.088
HHV8	HHV6	H	C	-0.471	0.466	-1.116, 0.173
HHV8	HHV6	M	C	0.011	1.000	-1.806, 1.828
HHV8	HHV6	C	C	-0.727	0.217	-1.619, 0.166
HSV	HHV6	H	C	-0.331	1.000	-1.056, 0.395
HSV	HHV6	M	C	-0.634	0.466	-1.500, 0.232
HSV	HHV6	P	C	-1.050	0.001	-1.922, -0.179
HSV	HHV6	C	C	-0.349	1.000	-0.974, 0.275
HHV8	HHV8	M	H	0.482	1.000	-1.363, 2.327
HHV8	HHV8	C	H	-0.255	1.000	-1.209, 0.698
HSV	HHV8	H	H	0.141	1.000	-0.848, 1.129
HSV	HHV8	M	H	-0.163	1.000	-1.085, 0.759
HSV	HHV8	P	H	-0.579	1.000	-1.507, 0.348
HSV	HHV8	C	H	0.122	1.000	-0.588, 0.831

HHV8	HHV8	C	M	-0.738	1.000	-2.686, 1.211
HSV	HHV8	H	M	-0.342	1.000	-2.216, 1.533
HSV	HHV8	M	M	-0.645	1.000	-2.746, 1.456
HSV	HHV8	P	M	-1.061	1.000	-2.998, 0.875
HSV	HHV8	C	M	-0.360	1.000	-2.202, 1.481
HSV	HHV8	H	C	0.396	1.000	-0.614, 1.406
HSV	HHV8	M	C	0.092	1.000	-1.023, 1.208
HSV	HHV8	P	C	-0.324	1.000	-1.444, 0.796
HSV	HHV8	C	C	0.377	1.000	-0.567, 1.321
HSV	HSV	M	H	-0.304	1.000	-1.284, 0.677
HSV	HSV	P	H	-0.720	0.466	-1.705, 0.265
HSV	HSV	C	H	-0.019	1.000	-0.803, 0.765
HSV	HSV	P	M	-0.416	1.000	-1.510, 0.677
HSV	HSV	C	M	0.285	1.000	-0.631, 1.200
HSV	HSV	C	P	0.701	0.368	-0.220, 1.622

Mean difference between shedding means comparing within-viruses or within cohorts. Comparisons were not made between cohorts across viruses (e.g., comparing mean difference of shedding of CMV in primary participants and EBV in HIV-positive mothers).
In the table, the following abbreviations were used: Ref. = reference, Comp. = comparator, P= children with primary infection, C= children with chronic infection, M=HIV-negative mothers, H=HIV-positive mothers.
